# Comparative Genomics and Specific Functional Characteristics Analysis of *Lactobacillus acidophilus*

**DOI:** 10.3390/microorganisms9091992

**Published:** 2021-09-20

**Authors:** Zheng Huang, Xingya Zhou, Catherine Stanton, Reynolds Paul Ross, Jianxin Zhao, Hao Zhang, Bo Yang, Wei Chen

**Affiliations:** 1 State Key Laboratory of Food Science and Technology, Jiangnan University, Wuxi 214122, China; 6190112150@stu.jiangnan.edu.cn (Z.H.); xingyazhouzxy@sina.com (X.Z.); zhaojianxin@jiangnan.edu.cn (J.Z.); zhanghao61@jiangnan.edu.cn (H.Z.); chenwei66@jiangnan.edu.cn (W.C.); 2 School of Food Science and Technology, Jiangnan University, Wuxi 214122, China; 3 International Joint Research Laboratory for Pharmabiotics & Antibiotic Resistance, Jiangnan University, Wuxi 214122, China; Catherine.stanton@teagasc.ie (C.S.); p.ross@ucc.ie (R.P.R.); 4 APC Microbiome Ireland, University College Cork, T12 K8AF Cork, Ireland; 5 Teagasc Food Research Centre, Moorepark, Fermoy, P61 C996 Cork, Ireland; 6 National Engineering Research Center for Functional Food, Jiangnan University, Wuxi 214122, China; 7 Wuxi Translational Medicine Research Center and Jiangsu Translational Medicine Research Institute Wuxi Branch, Wuxi 214122, China

**Keywords:** *Lactobacillus acidophilus*, comparative genomics, CRISPR-Cas, bacteriocin, antibiotic resistance

## Abstract

*Lactobacillus acidophilus* is a common kind of lactic acid bacteria usually found in the human gastrointestinal tract, oral cavity, vagina, and various fermented foods. At present, many studies have focused on the probiotic function and industrial application of *L. acidophilus*. Additionally, dozens of *L. acidophilus* strains have been genome sequenced, but there has been no research to compare them at the genomic level. In this study, 46 strains of *L. acidophilus* were performed comparative analyses to explore their genetic diversity. The results showed that all the *L. acidophilus* strains were divided into two clusters based on ANI values, phylogenetic analysis and whole genome comparison, due to the difference of their predicted gene composition of bacteriocin operon, CRISPR-Cas systems and prophages mainly. Additionally, *L. acidophilus* was a pan-genome open species with a difference in carbohydrates utilization, antibiotic resistance, EPS operon, surface layer protein operon and other functional gene composition. This work provides a better understanding of *L. acidophilus* from a genetic perspective, and offers a frame for the biotechnological potentiality of this species.

## 1. Introduction

*Lactobacillus acidophilus*, a Gram-positive bacterium with low GC content (34–37%), belongs to phylum Firmicutes, class Bacilli, order Lactobacillales, Family Lactobacillaceae, and Genus *Lactobacillus*. *L. acidophilus*, originally isolated from the infant feces in the 1900s and mostly found in the human gastrointestinal tract, oral cavity, vagina, and various fermented foods. Additionally, NCFM (a typical *L. acidophilus* strain) is one of the most well commercially and clinically well-researched probiotics. Since it was isolated, it has been studied and found to have a variety of beneficial properties for gastrointestinal and general health. Alleviating inflammatory bowel disease through reducing cytokines is the most well-known benefit of *L. acidophilus* [1,2,3]. Additionally, other health-associated functions of *L. acidophilus* have also attracted much attention, such as in alleviating cancer [4,5], regulating immunity [6,7], reducing cholesterol [8], and relieving diarrhea [9]. According to recent reports, it had been found that *L. acidophilus* could directly or indirectly interfere with the intestinal microbiota or host metabolism by some active substances, such as extracellular polysaccharides (EPS), surface layer protein (SLP) and bacteriocin.

With the development of high-throughput sequencing technology and comparative genomics approaches, numerous reports focused on genomes of lactobacilli, such as *Lacticaseibacillus* *rhamnosus* [10], *Lacticaseibacillus* *paracasei* [11], *Limosilactobacillus* *reuteri* [12,13], *Ligilactobacillus* *salivarius* [14], *Limosilactobacillus* *fermentum* [15], *Limosilactobacillus* *mucosae* [16], and *Ligilactobacillus* *ruminis* [17]. These studies used comparative genomics methods to reveal general genome characteristics, phylogenetic relationships and functional genes related to niche adaptation and probiotic activity of different strains, which let researchers have a new understanding of the metabolic capabilities and function roles of *lactobacilli* at the genetic level.

Although *L. acidophilus* possesses many probiotic characteristics, for instance, *L. acidophilus* NCFM, ATCC 4356 and ATCC 53544 were well-known probiotics and had been genomic sequenced; rare research on comparative genomics of this species has been reported [18,19].

In current researches, the probiotic functions of different *L. acidophilus* strains showed both similarities and differences which might be resulted from the diversification of *L. acidophilus*. Analysis of comparative genomes is possible to excavate the diversification of this species to expose the correlation between genotypes and phenotypes, and to predict the potential probiotic functions for new *L. acidophilus* isolates strains based on past researches. Hence, the purpose of this study was to sequence more genomes of *L. acidophilus* and to carry out comparative genomics analyses. These works will consolidate the foundation for future exploration of *L. acidophilus*, especially some typical physiological characteristics and synthetic beneficial metabolites.

## 2. Materials and Methods

### 2.1. Bacterial Strains Culturing and Genome Sequencing

Eleven strains of *L. acidophilus* were isolated from healthy human feces from different regions of China (Table 1), then 16S rRNA genes were sequenced for species identification. Additionally, *L. acidophilus* CCFM137 is another strain deposited at the Culture Collection of Food Microorganisms in Jiangnan University, Wuxi, China (CCFM). These 12 determined *L. acidophilus* strains were cultured with de Man, Rogosa and Sharpe (MRS) medium in an anaerobic workstation for 24 h [20] The draft genomes of these 12 strains were sequenced by Majorbio BioTech Co. (Shanghai, China), and SOAPde novo and GapCloser were used to assemble and fill the reads of draft genomes [21]. Additionally, another thirty four publicly available genomes of *L. acidophilus* from the National Centre for Biotechnology Information (NCBI) (https://www.ncbi.nlm.nih.gov/) (accessed on 15 August 2021) were used for comparison (Table 1).

### 2.2. The Average Nucleotide Identity (ANI) Values and Phylogenetic Analyses

The ANI values of any two genomes were calculated through a python script (https://github.com/widdowquinn/pyani) (accessed on 19 January 2021)and the resulting matrix was clustered and visualized using Seaborn, a Python data visualization library [22].

### 2.3. Pan-Genome and Core-Genome Analysis

Pan-genome and core-genome calculation of the *L. acidophilus* was performed using PGAP-1.2.1 [23]. Curve fitting of the pan-genome and core-genome were performed using a power-law regression based on Heaps’ law and an exponential regression model, respectively [24,25]. PanGP software was used to conduct fitting and visualize the core- and pan-genomes [26]. Venn diagram of core-genome and specific genome of those strains was made by using Orthomcl software [27]. 

### 2.4. Phylogenetic Comparison

Orthologous sequences of those forty-six strains were extracted by Orthomcl-v2.0.9 software for clustering orthologous genes [28]. A phylogenetic tree was constructed using the neighbor-joining (NJ) algorithm with default parameters in MEGA 7.0 software [29], and the phylogenetic tree was decorated with Evolgenius (http://www.evolgenius.info/evolview/) (accessed on 10 February 2021) [30].

### 2.5. Whole Genome Comparison

Genome wide visualization of coding sequences identity among all strains was used BLAST ring image to perform generator (BRIG). Additionally, *L. acidophilus* ATCC33323 was taken as a reference genome [31].

### 2.6. Genotype Analysis of Carbohydrate Metabolism

Those *L. acidophilus* genomes were annotated by HMMER-3.1, and the carbohydrate active enzymes were analyzed by Carbohydrate Active Enzymes Database7 (CAZy) (http://www.cazy.org/) (accessed on 10 February 2021) [32,33]. Additionally, *L. acidophilus* strains were clustered by software HEMI version 1.0.

### 2.7. Genotype Analysis of Antibiotic Resistance

The resistance gene identifier (RGI) software based on a comprehensive antibiotic resistance database (CARD) was used to analyze the antibiotic resistance of all *L. acidophilus* strains [34].

### 2.8. Prediction of the EPS and Surface Layer Protein Gene Operon

The sequences of those *L. acidophilus* strains were aligned with the EPS-encoding operon by the basic local alignment search tool (BLAST) program [35]. The presence of genes was determined based on the alignment fragment size and identity [36]. Easy sequencing in PostScript (ESPript) online program was used to display the aligned sequences [37].

### 2.9. Prediction of Bacteriocin Operon

The genes for potential bacteriocin operons were mined by using BAGEL4 webserver [38]. Additionally, BLASTP was used to analyze the domains of bacteriocin against the non-redundant protein databases created by BLASTP based on the National Center for Biotechnology Information (NCBI).

### 2.10. Identification of CRISPR-Cas Systems and Prophage

The CRISPRCasFinder with default parameters was used to identify the clustered regularly interspaced short palindromic repeats (CRISPR) regions and CRISPR-associated (Cas) proteins [39]. The PHASTER (PHAge Search Tool Enhanced Release) webserver was used to identify and annotate the prophages within all the strains [40].

### 2.11. Ethics Statement

This study was approved by the Ethics Committee in Jiangnan University, China (SYXK 2012-0002). All the fecal samples from healthy persons were for public health purposes and these were the only human materials used in the present study. Written informed consent for the use of their fecal samples was obtained from the participants or their legal guardians. All of them conducted health questionnaires before sampling and no human experiments were involved. The collection of the fecal sample had no risk of predictable harm or discomfort to the participants.

## 3. Results

### 3.1. Genome Characteristics of L. acidophilus

In this study, 11 strains of *L. acidophilus* were isolated from human feces in different areas in China (Table 2), and in total 46 genomes were compared in this study (Table 1). The genome size of all the strains ranged from 1.95 Mb to 2.09 Mb, and the average size was 1.98 Mb. The average G + C content and number of coding sequences was 34.66% and 1780, respectively. Compared with the strains isolated from humans, those from fermented foods and commercial probiotic products possessed more coding sequences.

### 3.2. Pan-Genome and Core-Genome of L. acidophilus

**Figure 1:**
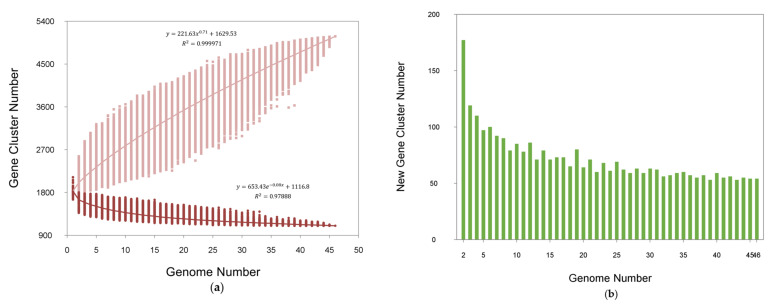
Pan-genome and core-genome of *L. acidophilus.* (**a**) Pan-genome plot is represented by the accumulated number of new genes against the number of genomes added. Core-genome plot is represented by the accumulated number of genes attributed to the core-genomes against the number of added genomes. (**b**) The new gene cluster number plot shows the changes in new genes of all strains genomes as the number of *L. acidophilus* strains increases.

**Figure 2:**
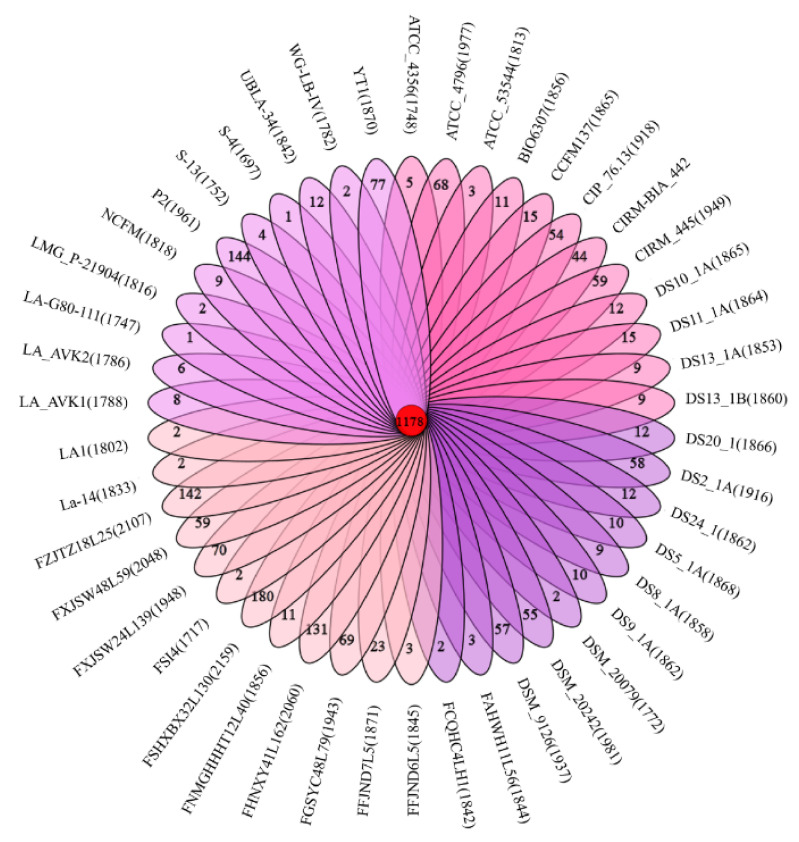
Venn diagram of *L. acidophilus*. The middle circle of the Venn diagram shows the number of homologous genes of these 46 *L. acidophilus*. Additionally, every branch shows the number of their unique genes.

Pan-genome is the general term for all the genes of a species. In this study, the genetic diversity of *L. acidophilus* was shown by the core- and pan-genome curves. Additionally, the curve presented an asymptotic trend (Figure 1a). The number of new gene cluster increments gradually decreased from 141 to 13 (Figure 1b). According to the regression equation of the pan-genome, its size increased infinitely as new genomes were added sequentially, which indicated the pan-genome of *L. acidophilus* could be considered as an open state, which meant that the pan-genome size of species increased with the number of sequenced genomes. With the equation of core-genome, there were approximately 1117 genes harbored in the core-genome of *L. acidophilus* (Figure 1a). The specific and homologous core genes were represented by the Venn diagram among all the 46 *L. acidophilus* strains, which showed that there were 1178 shared genes among all the strains. Additionally, the number of distinctive genes for each *L. acidophilus* strain ranged from 1 to 180 (Figure 2).

### 3.3. ANI, Phylogenetic Analyses and Whole Genome Comparison of L. acidophilus

**Figure 3:**
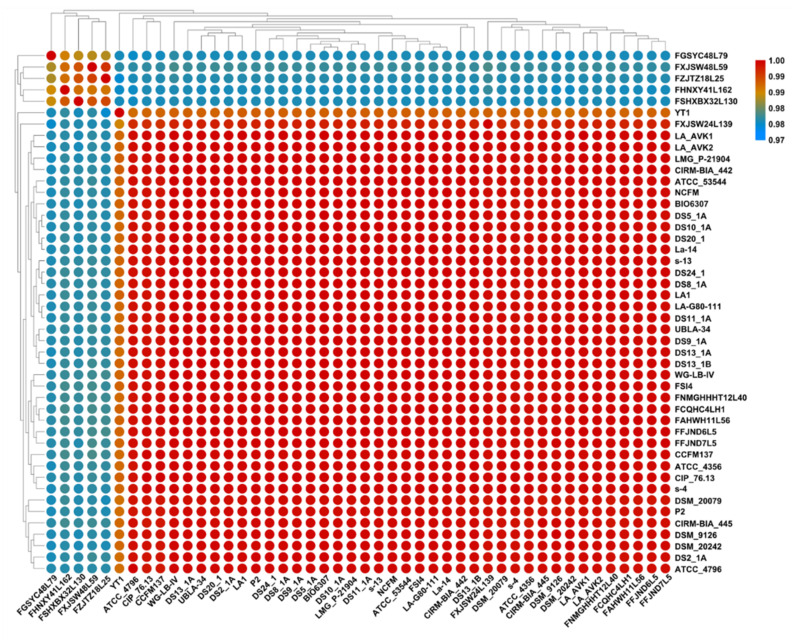
ANI Heatmap of *L. acidophilus*. Proposed species cut-off boundary is above 97%, showing identity within these strains.

ANI analysis is a common standard for species classification and clustering. A previous study found that in most lineages, there was a clear dividing line of ANI within and between species, in which the ANI value of the same species was higher than 95%, and the ANI value of different species was less than 95% [41]. The results showed that all the 46 strains with ANI values above 97% belonged to *L. acidophilus*. However, all the strains were significantly divided into two regions, in which a small region consisted of FZJTZ18L25, FSHXBX32L130, FGSYC48L79, FHNXY41L162 and FXJSW28L59 and a large region consisted of all the other 41 strains (Figure 3).

**Figure 4:**
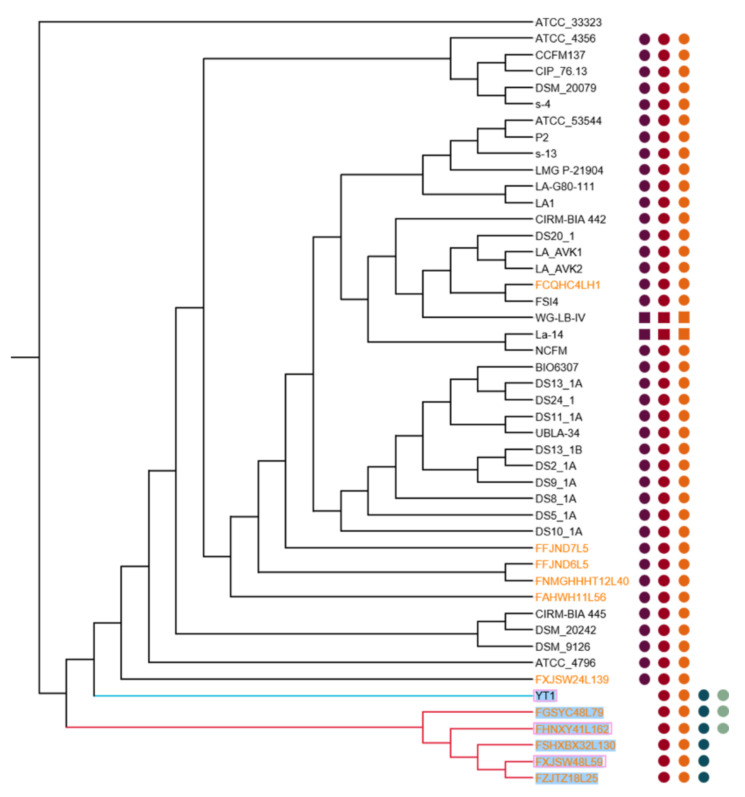
Phylogenetic analysis of *L. acidophilus*. Phylogenetic tree showing the relationship among 46 *L. acidophilus* strains with *L. gasseri* ATCC33323 as outgroup. The tree was grouped into three clades signed by different colors of lines (red represented clade A, blue represented clade B and black represented clade C). The dots represented strains that contained the operon of bacteriocins. In specific, dark purple dots represented Acidocin_J1132_beta_peptide_N-terminal, claret dots represented Bacteriocin_helveticin_J, orange dots Enterolysin_A, mazarine dots Helveticin-J, green dots Lanthipeptide. Additionally, circular dots represented one bacteriocin gene operon while square dots represented two. Orange font represented strains isolated in this study, black represented obtained from NCBI. Pink frames represented strains that contained a complete CRISPR/Cas system. Wathet highlight represented strains contained intact prophage.

A phylogenetic tree was performed based on the homologous genes of 46 *L. acidophilus* genomes in order to analyze the phylogenetic relationship of *L. acidophilus* strains (Figure 4). The phylogenetic tree revealed that all the 46 *L. acidophilus* strains formed three branches, which were rooted by *L. gasseri* ATCC33323 as an outgroup, and the *L. acidophilus* strains were divided into three clades (clade A, B and C). Clade A and clade B included six strains which could synthesize Helveticin-J, while all the strains in clade C predictably had the Operon Acidocin_J1132_beta_peptide_N-terminal. Interestingly, the composition of clade a on phylogenetic tree was the same as the small region in the ANI heatmap.

**Figure 5:**
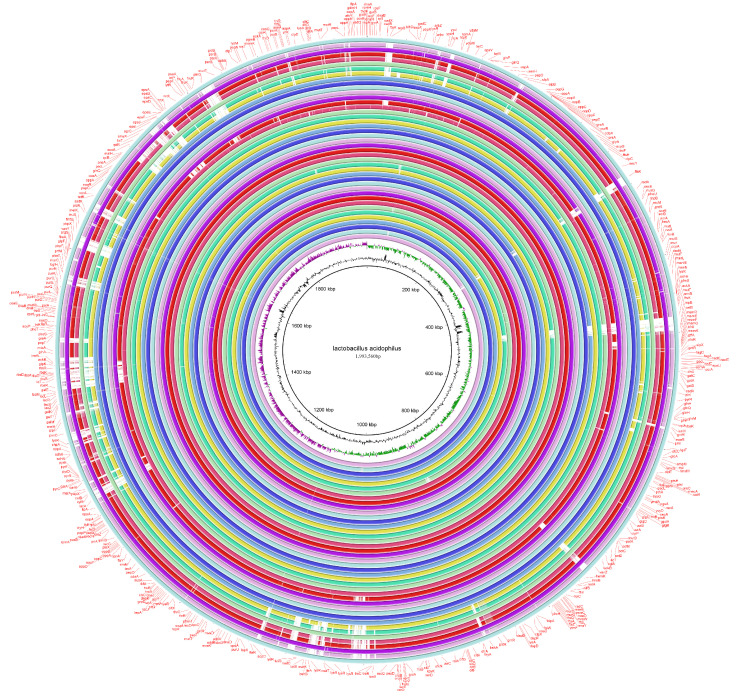
The whole genome comparison of *L. acidophilus*.

With *L. acidophilus* NCFM as a referenced genome, the whole genomes of all the 46 strains were analyzed through BRIG software. Most strains had no significant difference in their genome composition, apart from the clade A and small region clustered strains FZJTZ18L25, FXJSW28L59, FSHXBX32L130, FHNXY41L162, FGSYC48L79 and YT1, whose genome sequences located at rings 2, 4, 5, 7, 8 and 14, counting from the outside in the ring image (Figure 5). Those six genetically similar strains had similar gene deletion, such as *ftsK*, *celB*, *repB* and *marR*, which encoded putative cell division proteins, cellobiose-specific PTS IIC, putative replication initiator proteins and transcriptional regulators, respectively. Additionally, some genes related to ribose encoding including *rbsB*, *rbsK* and *rbsR* were absent.

### 3.4. Genotype Analysis for Carbohydrates Utilization in L. acidophilus

**Figure 6:**
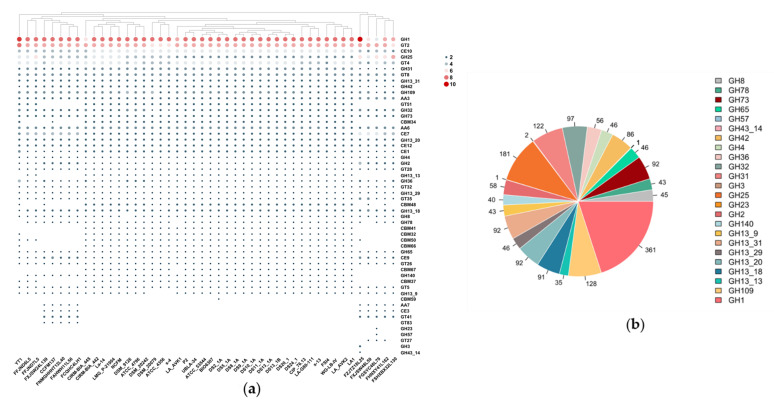
Carbohydrate utilization families identified in *L. acidophilus.* (**a**) Heatmap showing the distribution of carbohydrates utilization families. (**b**) Pie chart indicating the number of each GH family identified.

In this study, the CAZy database was utilized to analyze the 46 *L. acidophilus* genomes in order to explore how those strains using different carbohydrates. The results revealed that there were 54 genes encoding predicted enzymes which could activate carbohydrates, including 24 glycoside hydrolases (GH) families, 12 glycosyl transferases (GT) families, three auxiliary activities (AA) families, 9 carbohydrate-binding modules (CBM) families and six carbohydrate esterases (CE). GH1 (β-glucosidase, EC 3.2.1.21), GH3 (β-glucosidase, EC 3.2.1.21), GH13_20 (α-amylase, EC 3.2.1.1), GH13_31 (α-amylase, EC 3.2.1.1), GH25 (lysozyme, EC 3.2.1.17), GH73 (lysozyme, EC 3.2.1.17), GT2 (cellulose synthase, EC 2.4.1.12), GT4 (sucrose synthase, EC 2.4.1.13), GT8 (lipopolysaccharide α-1,3-galactosyltransferase, EC 2.4.1.44), GT41 (lipopolysaccharide α-1,3-galactosyltransferase, EC 2.4.1.44), GT51 (murein polymerase, EC 2.4.1.129), AA3 (cellobiose dehydrogenase, EC 1.1.99.18), AA6 (1,4-benzoquinone reductase, EC. 1.6.5.6), CE7 (acetyl xylan esterase, EC 3.1.1.72), CE10 (arylesterase, EC 3.1.1.-) and CE12 (pectin acetylesterase, EC 3.1.1.-) were distributed among all the *L. acidophilus* strains, while the remaining families had different distribution in those strains (Figure 6a).

In carbohydrate metabolism, glycosyl hydrolases are the key enzymes. Among all the 24 predicted GH families, 15 of them were present in each strain. Additionally, those 15 families were involved in the metabolism of common carbohydrates, such as glucose, galactose, fructose, sucrose, starch and maltose in the human diet. In all GH families, GH3 (β-glucosidase, EC 3.2.1.21), GH23 (lysozyme type G, EC 3.2.1.17), GH43_14 (β-xylosidase, EC 3.2.1.37) and GH57 (α-amylase, EC 3.2.1.1) were the least four GH families (Figure 6b).

### 3.5. Genotype Analysis for Antibiotic Resistance of L. acidophilus

**Figure 7:**
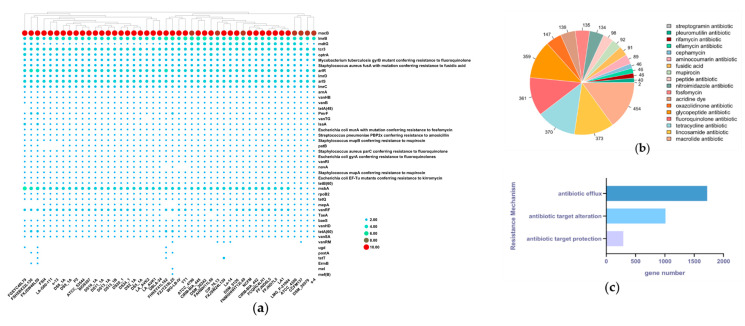
Antibiotic resistance gene of *L. acidophilus.* (**a**) Heatmap showing number of antibiotic resistance gene. (**b**) Pie chart indicating the classes of antibiotics. (**c**) Bar plot showing gene number of different kinds of resistance mechanism.

The number of different genes in all *L. acidophilus* was analyzed and annotated by RGI software. Genetically, *macB* and *lmrB* were the top two genes of antibiotic resistance in those strains whose numbers were nine to ten and four to five, respectively, while the number of other genes was less than four. In contrast, *vanRM*, *ugd*, *poxtA*, *telT*, *ErmB*, *mel* and *mef(B)* genes were the least seven genes related to antibiotic resistance in *L. acidophilus* (Figure 7a).

With all the genes of antibiotic resistance, there were 18 different classes of antibiotic (acridine dye, aminocoumarin antibiotic, cephamycin, elfamycin antibiotic, fluoroquinolone antibiotic, fosfomycin, fusidic acid, glycopeptide antibiotic, lincosamide antibiotic, macrolide antibiotic, mupirocin, nitroimidazole antibiotic, oxazolidinone antibiotic, peptide antibiotic, pleuromutilin antibiotic, rifamycin antibiotic, streptogramin antibiotic and tetracycline antibiotic) resistant genes in *L. acidophilus*. Fluoroquinolone, glycopeptide, lincosamide, macrolide and tetracycline antibiotics were the most five classes of antibiotics that *L. acidophilus* could tolerate. There were more than 300 relevant genes of these five antibiotics distributed in *L. acidophilus*. (Figure 7b). For the resistance pattern, *L. acidophilus* could resist antibiotics through antibiotic efflux, antibiotic target alteration and antibiotic target protection (Figure 7c).

### 3.6. Comparative Analysis of Functional Gene Composition of L. acidophilus

**Figure 8:**
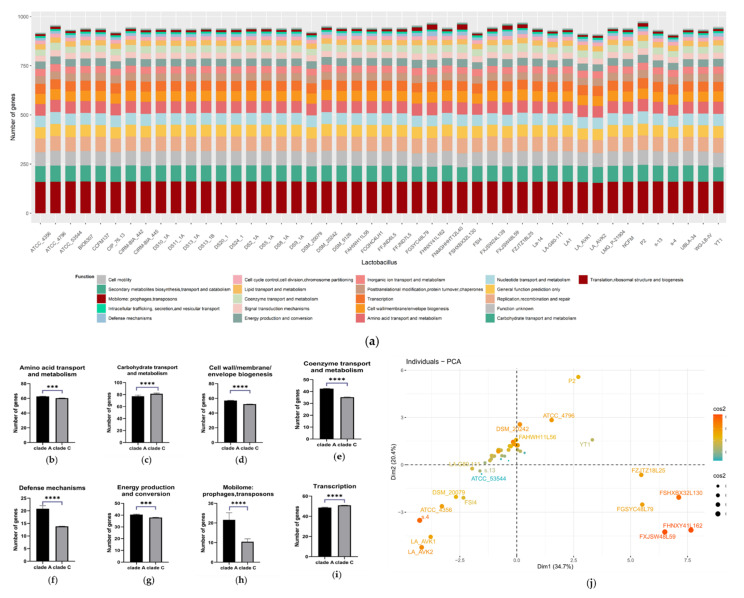
COG analysis of *L. acidophilus*. (**a**) Composition of COG function categories. (**b**–**i**) Number of amino acid transport genes; carbohydrate transport and metabolism genes; cell wall/membrane/envelope biogenesis genes; coenzyme transport genes; defense mechanisms genes; energy production genes; energy production genes; mobilome (prophage and transposons) genes; transcription genes in clade A and C *L. acidophilus* strains. ***: *p* < 0.001, ****: *p* < 0.0001. All data are presented as mean ± SEM. (**j**) PCA analysis of composition of COG function categories in different *L. acidophilus* strains.

The Clusters of Orthologous Groups (COG) database is a database for identifying orthologous genes. After COG comparison and analysis of the difference in the number of functional genes, the results showed that there was a significant difference in some functional genes, such as amino acid transport genes, carbohydrate transport and metabolism genes, cell wall/membrane/envelope biogenesis genes, coenzyme transport genes, defense mechanisms genes, energy production genes, mobilome (prophage and transposons) genes and transcription genes in *L. acidophilus* (Figure 8a–i). Furthermore, from PCA analysis, most *L. acidophilus* strains were clustered together except a small clustered group consisted of five strains including YT1, FZJTZ18L25, FSHXBX32L130, FGSYC48L79, FHNXY41L162 and FXJSW28L59, which was consistent with the phylogenetic tree clustering result (Figure 8j).

### 3.7. Prediction of the EPS Operon in L. acidophilus

**Figure 9:**
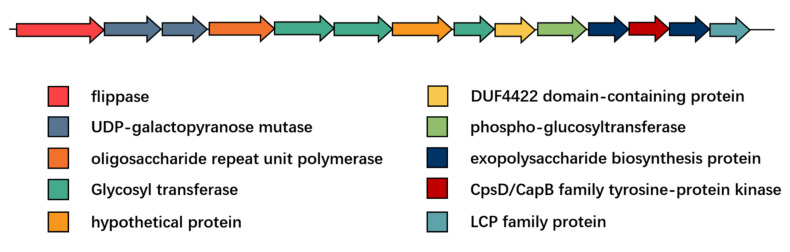
EPS gene cluster in *L. acidophilus NCFM*.

**Figure 10:**
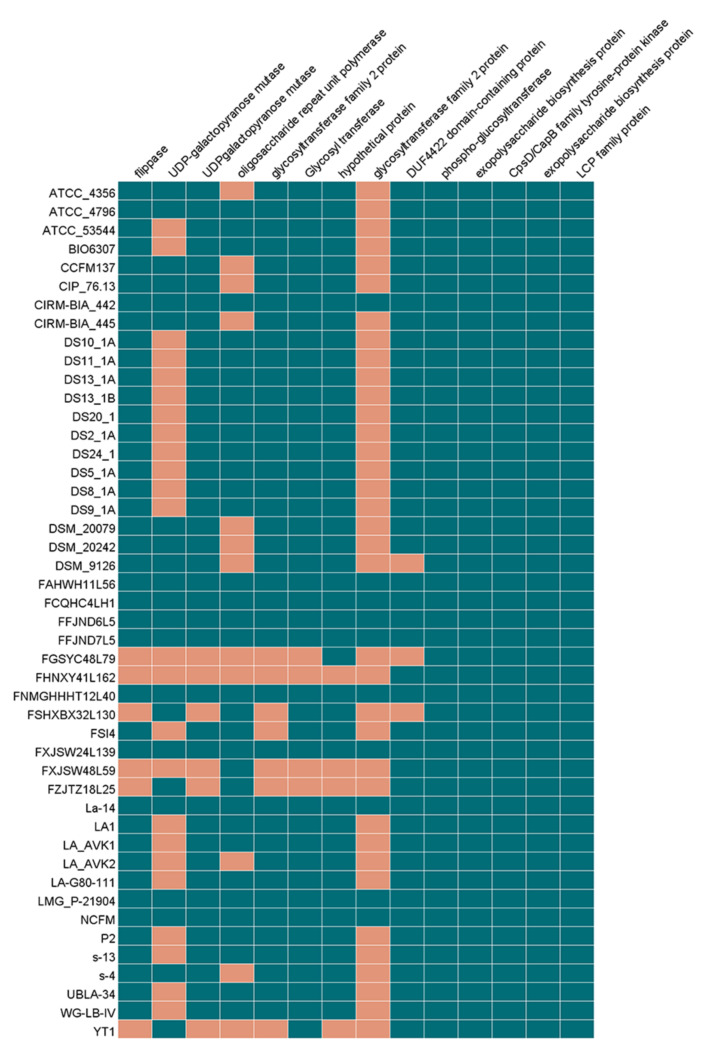
Heatmap of EPS gene cluster in *L. acidophilus*. Black green regions represented the specific genes exist in strains. The orange regions represented the specific genes do not exist in strains.

EPS is a key structural and functional composition in *L. acidophilus*. To figure out whether the newly genome-sequenced *L. acidophilus* could produce EPS, Orthomcl software and BlastN were used to predict the gene operon of EPS. All the protein sequences of EPS gene clusters were integrated together according to NCFM after Orthomcl analysis. For NCFM, the EPS gene cluster mainly composed of 14 genes including the highly conserved proteins LCP family protein (EpsA), exopolysaccharide biosynthesis protein (EpsB), CpsD/CapB family tyrosine-protein kinase (EpsC), exopolysaccharide biosynthesis protein (EpsD), phospho-glucosyltransferase (EpsE), DUF4422 domain-containing protein (EpsF), flippase (EpsI), UDP-galactopyranose mutase (EpsJ) and six variable proteins representing glycosyltransferases and polysaccharide polymerases (Figure 9). Among all the 46 *L. acidophilus* strains, there were only nine strains (CIRM_BIA_442, FAHWH11L56, FCQHC4LH1, FFJND6L5, FNMGHHHT12L40, FXJSW24L139, La-14 and NCFM) consisting of whole EPS-producing operons (Figure 10).

### 3.8. Prediction of the Surface Layer Protein Operon in L. acidophilus

**Figure 11:**
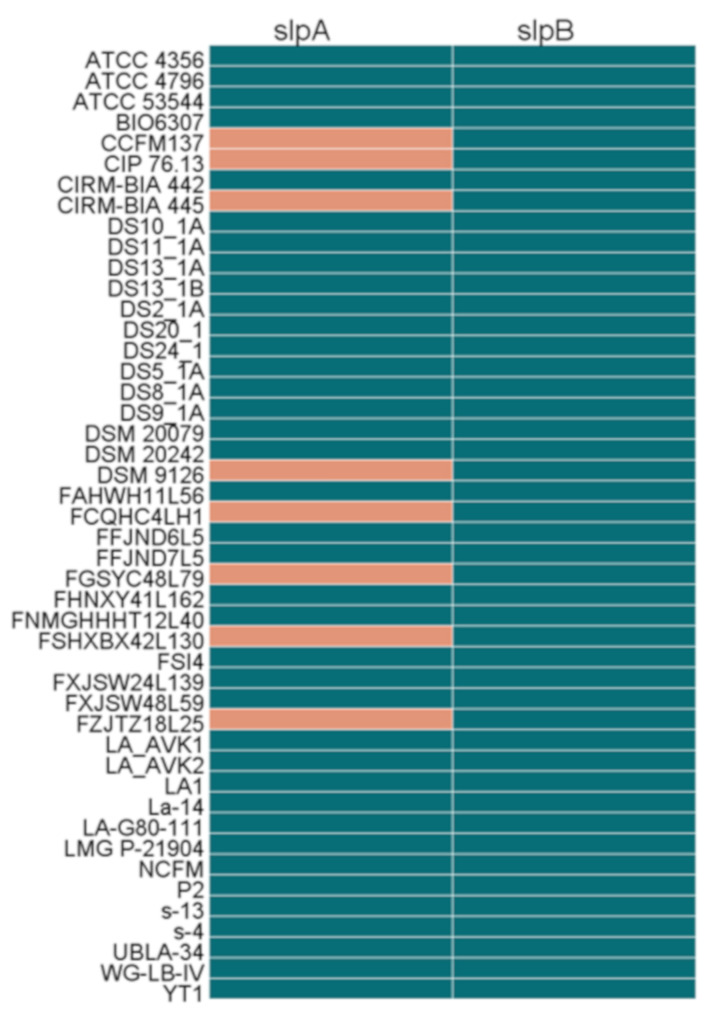
Heatmap of surface layer protein gene in *L. acidophilus*. Black green regions represented the specific genes exist in strains. The orange regions represented the specific genes do not exist in strains.

Surface layer protein was one of the contents that could possess the biological properties of *L. acidophilus*. To explore whether the *L. acidophilus* newly isolated in this study could produce surface layer protein, the homologous gene operon that could encode this protein was predicted by Orthomcl software and BlastN. In most *L. acidophilus* strains, there were three independent genes relevant to surface layer protein, *slpA* could encode for the pore-forming S-layer protein SlpA (44,884 Da), and other two genes were identified as absent genes called *slpB* (43,636 Da), which could encode hypothetical SLAP domain-containing protein SlpB with 53% similarity to SlpA in the N-terminal and middle parts and only one amino acid residue difference in the C-terminal if this silent gene could express. With homologous gene analysis and multiple alignments of protein sequences, eight *L. acidophilus* strains including CCFM137, CIP_76.13, CIRM_BIA_445, DSM_9126, FCQHC4LH1, FGSYC48L79, FSHXBX32L130 and FZJTZ18L25 could not encode SlpA protein while other thirty-eight strains could encode it predictively. For putative protein SlpB, each strain had the corresponding genes (Figure 11).

### 3.9. Prediction of the Bacteriocin Operon in L. acidophilus

**Figure 12:**
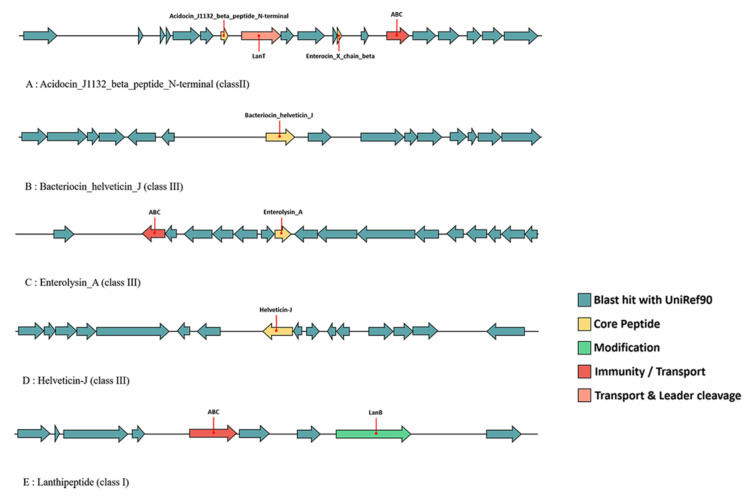
Predicted bacteriocin gene operon in *L. acidophilus*.

The potential operon of bacteriocin in the 46 *L. acidophilus* strains was mined using the BAGEL4 webserver in this study. The results showed that in total five different bacteriocins including acidocin_J1132_beta_peptide_N-terminal (6.2), bacteriocin_helveticin_J (6.3), enterolysin_A (64.3), helveticin-J (70.3) and lanthipeptide were predicted (Figure 4). In general, the bacteriocins synthesized by *L. acidophilus* covered all three classes, of which class II was the main one. Each strain was predicted that they could synthesize bacteriocin_helveticin_J and enterolysin_A, while only six and three strains could synthesize helveticin-J and lanthipeptide, respectively. Acidocin_J1132_beta_peptide_N-terminal was another common bacteriocin, which was identified in forty strains. Among all the 46 *L. acidophilus*, La-14 and WG-LB-IV were two special strains which could synthesize acidocin_J1132_beta_peptide_N-terminal, bacteriocin_helveticin_J and enterolysin_A and with two operons, respectively, while the other 44 strains only possessed one operon. Each bacteriocin gene operon in *L. acidophilus* contained its core peptides (Figure 12).

### 3.10. Prediction of Prophages and CRISPR-Cas Systems in L. acidophilus

The prophages of *L. acidophilus* were predicted by PHASTER, and only six strains (FGSYC48L79, FHNXY41L162, FSHXBX32L130, FXJSW48L59, FZJTZ18L25 and YT1) contained intact prophages sequences (Table 3). Among them, FGSYC48L79 and FHNXY41L162 carried out two prophages, FSHXBX32L130 carried out three prophages, while FXJSW48L59, FZJTZ18L25 and YT1 only possessed one prophage, respectively. Interestingly, FGSYC48L79, FHNXY41L162, FSHXBX32L130, FXJSW48L59 and FZJTZ18L25 were clustered into same clade (clade A), while YT1 was clustered into their neighboring clade (clade B) on the phylogenetic tree (Figure 4).

All the 46 genome sequences were uploaded to CRISPRCasFinder and predicted by orphan CRISPRs, which had no Cas proteins. Only three strains (FHNXY41L162, FXJSW48L59 and YT1) had complete CRISPR-Cas systems and all of them contained intact prophages (Figure 4). However, none of their spacers in CRISPR could correspond to the prophages’ sequences within them.

## 4. Discussion

*L. acidophilus* is a kind of lactic acid bacteria that has been widely used in industry for a long time and has excellent health-associated benefits. In previous studies, *L. acidophilus* was reported as a member of *L. acidophilus* group for phylogeny and comparative genome analysis [19]. However, due to the limitation of sequencing technology development and genome predictive analysis tools at that time, the results of gene prediction and annotation have an era limitation. With the development of sequencing technology and bioinformatics tools, researchers have the possibility of deeper analyzing the genomes. In this study, for the first time, *L. acidophilus* was analyzed to figure out the differences among strains through the comparative genomics approaches, including generally genomic characteristics, phylogenetic analysis, and prediction of some functional genes.

The average G + C content of *L. acidophilus*, one of the standard features in bacterial taxonomy, was 34.66%, and this value could reflect the genetic relationship in evolution to some extent. This value was consistent with that in Bergey’s Manual of Systematics of Archaea and Bacteria [42], but it was lower compared to other lactobacilli (with more than 40% G + C content). Additionally, its average size of genome was 1.98 Mb with ~1800 CDSs. Combined with COG analysis, the core genes of those strains were mainly carbohydrate transport and metabolism, defense mechanisms, translation, ribosomal structure and biogenesis and other basic functions.

The pan-genome consists of the core genome and the dispensable genome. The genetic plasticity and environmental adaptation potential of a species could be indicated from the relative size and content of the pan-genome. In the context of rapid development of high-throughput sequencing technology, it is convenient and quick to generate the whole genome for a strain of bacteria. Additionally, it is valuable to sequence its pan-genome to estimate the size of the entire gene repertoire and the diversity of this species [25]. Through the prediction of mathematical models, even if hundreds of genomes of each species were sequenced, there would be newly discovered genes in subsequent studies [43]. However, compared with the pan-genome of other lactobacilli, such as *Limosilactobacillus* *mucosae* (8100 genes) [16], *Ligilactobacillus* *ruminis* (10,000 genes) [17], *Lacticaseibacillus rhamnosus* (8200 genes) [44] and *L. gasseri* (6500 genes) [45], the pan-genome of *L. acidophilus* was smaller (5200 genes) and open. From the perspective of habitat, with *Limosilactobacillus* *mucosae* as an example, in addition to human feces and fermented dairy products, they had different habitat sources such as piglets, dogs and cattles. Their wide habitat range made them have larger gene pools for lateral gene transfer. The source of *L. acidophilus* used in this study was not sundry, and those strains were isolated from human feces and fermented products mainly. Hence, the open and narrow pan-genome status of *L. acidophilus* indicated that its genetic diversity could be further enriched, and it also had the ability to continue to adapt to various ecological niches. The similar inference had also been mentioned in previous study of *Streptococcus* [46]. From another point of view, the pan-genome of *L. acidophilus* may be due to its relatively stable intestinal niche, the small intestine environment, which was different from other lactobacilli colinized in the large intestine, and the living conditions abundance of the microflora of small intestine were lower than that in the large intestine, therefore, the living environment and niche for *L. acidophilus* was more stable, and there were fewer external changes and interferences, which contributed to that *L. acidophilus* did not need a wider pan-genome range to adapt to its niche.

ANI has been used to substitute DNA-DNA hybridization as the gold standard for prokaryotic species genetical circumscriptions [22]. Based on ANI, some phenotypically and genotypically closely related species, such as *L. casei*, *Lacticaseibacillus* *paracasei* and *Lacticaseibacillus rhamnosus*, that were difficult to distinguish on taxonomic level, had a new method to be identified [47,48,49]. Therefore, this study followed previous methods and carried out ANI analysis on *L. acidophilus* with the aim to explore the diversity of the species and the existence possibility of subspecies. All the 46 strains with ANI value above 97% were classified into two clusters, with five strains showing an ANI value over 99%, and the other group consisted of 41 strains including *L. acidophilus* NCFM with an ANI range 97–98% compared with another group. The ANI value of *L. acidophilus* was relatively narrow, compared with *Limosilactobacillus* *mucosae* (95.5%) [16] and *Ligilactobacillus* *ruminis* (96%) [17], representing that the proportion of variable genes was less, and the diversity was not rich. Combining the results of pan-genome analysis, it was speculated that *L. acidophilus* may not need many genes to adapt to different niches. Phylogenetic analyses and whole genome comparison of *L. acidophilus* were performed to further mine the relationship of the two classified groups. Interestingly, the five strains gathered in ANI analysis were also clustered in the branch on the phylogenetic tree. Meanwhile, they had similar levels of gene deletion. It was speculated that those five strains may be potential subspecies in *L. acidophilus*. However, due to the small sample size, this hypothesis still needs further verification.

For bacteria living in the intestine, their ability to use nutrients partly determined their ability to reside and survive in the intestine. In different nutrient environments, the carbohydrate utilization genes in *L. acidophilus* will be differentially expressed [50]. In silico, there were 24 GH series involving carbohydrate metabolism showed that GH1 accounted for a relatively high proportion of GH, showing that the main carbon source of *L. acidophilus* was glucose. Additionally, the number of genes in carbohydrate transport and metabolism in COG analysis showed that there was a significant difference between clade A and C, which meant that in *L. acidophilus* there might be two branches with different carbohydrate utilization.

Similar to other probiotics, *L. acidophilus* can easily obtain different antibiotic resistance genes in the intestine through mobile genetic elements. If it is to be added to food as a food additive, it needs to undergo safety verification [51]. In this study, predictions of antibiotic resistance genes have been made for each strain. Additionally, *L. acidophilus* has the most resistance genes of fluoroquinolone, glycopeptide, lincosamide, macrolide and tetracycline, which was consistent with previous reports [52,53,54,55].

The lactobacilli EPS had the effect of regulating intestinal immunity. Additionally, *L. acidophilus* NCFM had been reported that its EPS induced genes expression related to immunity both in vitro and in vivo [56]. Additionally, the EPS (LA-EPS-20079) of *L. acidophilus* DSM20079 had been proved that exerted a direct cytotoxic action on the tumors cells in addition to stimulating the immune response and inflammatory pathway [4]. Referring to NCFM as the standard [2005NCFM], the EPS cluster showed high synteny to EPS cluster of *L. gasseri* [45], *L. johnsonii* [57], *Lacticaseibacillus casei* [58] and *streptococci* [59]. The relationship between EPS gene clusters and EPS synthesis in *Lacticaseibacillus casei* was investigated, and that glucose-1-phosphate thymidyltranseferase gene (LC2W_2179), uncharacterized EPS biosynthesis protein (LC2W_2188), and EPS biosynthesis protein (LC2W_2189) were related to EPS biosynthesis. According to these results, *epsB* (exopolysaccharide biosynthesis protein gene) and *epsD* (exopolysaccharide biosynthesis protein gene) may exert the same effect in *L. acidophilus* NCFM. Although not all the *L. acidophilus* strains had a complete EPS gene cluster composing of 14 genes, similar to NCFM, each strain had *epsB* and *epsD*, indicating that all the 46 strains may have the potential to synthesize EPS.

Surface layer protein A (SlpA) of *L. acidophilus* is a key factor in probiotic–host crosstalk and could trigger immunomodulation in the host [2,60,61]. Different from EPS, SlpA is a protein regulated by a single gene. There were eight out of all the *L. acidophilus* strains that possessed no SlpA encoding gene. However, the expression of surface layer protein is related to some environmental factors, such as anaerobic conditions [62], bile salt concentrations [63], mucin, pancreatin and pH [64]. Therefore, whether *L. acidophilus* could produce SlpA or not need more experiments to verify.

Since the discovery of antimicrobials, compounds that could kill or inhibit the growth of bacteria, human life expectancy was improved. Nevertheless, antibiotic resistance has become a major threat. The bacteriocin derived from the intestinal microflora has shown great potential in maintaining intestinal homeostasis and biological control of pathogenic bacteria [65]. Bacteriocin production is one of the characteristics of *L. acidophilus*. For example, *L. acidophilus* JCM 1132 produced acidocin J1132 that had a narrow inhibitory spectrum [66], in addition, TK9201 produced acidocin A [67], DSM20079 generated acidocin D20079, and PNW3 produced a bacteriocin predicted to be helveticin J [68]. Additionally, a novel class III bacteriocin gene (NX371) was mined by bioinformatic analysis in *L. acidophilus* NX2-6, which had 98.15% homology of helveticin J [69]. Combining the results of previous researches and bioinformatics predictions in this research, it can be inferred that, *L. acidophilus* mainly produce a variety of class II (acidocin) and class III (helveticin J) bacteriocins. Acidocin was the most common bacteriocin in *L. acidophilus* [66,69,70,71,72,73,74,75]. However, in the prediction of bacteriocin operon, six strains did not have the gene cluster of acidocin-like bacteriocin. Coincidentally, all of them had hypothetical operon of helveticin-J. It was inferred that they may inhibit other bacteria by encoding helveticin-J instead of acidocin-like bacteriocin. Gene cluster of enterolysin A was predicted in all strains, and there were no relevant reports that it can be purified from *L. acidophilus*. Enterolysin A, an antimicrobial protein that could inhibit the growth of specific bacteria, was purified from an *Enterococcus faecalis* LMG 2333 [76]. *Enterococcus* has a high tendency to acquire and express new determinants of resistance, and the acquired resistance can then be transferred to other bacteria through mobile genetic elements [77]. That may be the reason why some *L. acidophilus* had the operon of enterolysin A. Lanthipeptide is not a typical bacteriocin in lactobacilli, but three *L. acidophilus* strains possessed the potentially synthetic genes.

In this study, only six strains carried out intact prophages sequences, and the number was very few compared to other lactobacilli [16,17,44,46]. Moreover, as the result of COG analysis, the number of prophages and transposons genes of those six strains clustered in clade A were more than twice as much as clade C. The reason could be due to the niche of *L. acidophilus*, which is the small intestine, a place where the living environment is much worse than the colon and cecum. This will result in not many prophages being able to exist in such a niche and *L. acidophilus* may not integrate the sequences of the prophages into their own genomes. Similarly, only three strains had complete CRISPR-Cas systems, which meant that they did not need the existence of CRISPR-Cas system, whose possession could be non-adaptive for strains [78] to resist foreign DNA invasion.

## 5. Conclusions

Based on ANI values, phylogenetic analysis and whole genome comparison, 46 *L. acidophilus* strains could be divided into two parts, which could be caused by the difference of predicted gene composition of bacteriocin operon, CRISPR-Cas systems and prophages mainly. In addition, predictably, genes related to carbohydrate utilization, EPS production, surface layer protein production, and antibiotic resistance of different *L. acidophilus* strains were all different. Hence, genome sequencing and genetic analysis enabled this research to deeply understand and exploit the biotechnology potential of *L. acidophilus*.

## Figures and Tables

**Table 1:** Source information, BioSample and general genome features of 46 *L. acidophilus* strains.

Strain	BioSample	Size(Mb)	GC(%)	CDS no.	Source
ATCC 4356	SAMN03105773	1.9567	34.6	1730	Human
ATCC 4796	SAMN00001471	2.0205	34.7	1747	Human
ATCC 53544	SAMN07357495	1.99191	34.7	1773	Human
BIO6307	SAMN12856535	1.96977	34.6	1790	Unknown
CCFM137	SAMN19655193	1.951495	34.58	1904	Human
CIP 76.13	SAMEA2272342	1.95182	34.6	1741	Human
CIRM-BIA 442	SAMEA2272381	1.98699	34.7	1787	Dairy product
CIRM-BIA 445	SAMEA2272655	2.00201	34.6	1782	Dairy product
DS10_1A	SAMN05583778	1.97072	34.6	1787	Commercial Probiotic Products
DS13_1A	SAMN05583782	1.96427	34.6	1780	Commercial Probiotic Products
DS13_1B	SAMN05583783	1.96393	34.6	1785	Commercial Probiotic Products
DS2_1A	SAMN05583785	1.98202	34.6	1812	Commercial Probiotic Products
DS20_1	SAMN06464087	1.96947	34.6	1795	Commercial Probiotic Products
DS24_1	SAMN06464090	1.96837	34.6	1790	Commercial Probiotic Products
DS5_1A	SAMN05583788	1.96883	34.6	1784	Commercial Probiotic Products
DS8_1A	SAMN05583791	1.96757	34.6	1787	Commercial Probiotic Products
DS9_1A	SAMN05583792	1.96901	34.6	1792	Commercial Probiotic Products
DSM 20079	SAMN06606133	2.00997	34.7	1760	Human
DSM 20242	SAMEA2272474	2.04786	34.7	1814	Unknown
DSM 9126	SAMEA2272239	1.99176	34.6	1790	Unknown
FAHWH11L56	SAMN19655182	1.975822	34.591	1881	Human
FCQHC4LH1	SAMN19655183	1.962741	34.569	1877	Human
FFJND6L5	SAMN19655184	1.960445	34.562	1878	Human
FFJND7L5	SAMN19655185	1.989652	34.581	1905	Human
FGSYC48L79	SAMN19655186	1.990393	34.662	1984	Human
FHNXY41L162	SAMN19655187	2.051142	34.733	2112	Human
FNMGHHHT12L40	SAMN19655188	1.992905	34.658	1894	Human
FSHXBX32L130	SAMN19655189	2.140788	34.893	2218	Human
FSI4	SAMN03274004	1.99197	34.7	1790	Fermented dairy product
FXJSW24L139	SAMN19655190	2.115801	34.647	2059	Human
FXJSW48L59	SAMN19655191	2.048808	34.753	2100	Human
FZJTZ18L25	SAMN19655192	2.115442	34.846	2170	Human
LA_AVK1	SAMN13198235	1.96274	34.6	1690	Unknown
LA_AVK2	SAMN13198280	1.96264	34.6	1690	Unknown
LA1	SAMN05631052	1.9912	34.7	1787	Fermented dairy product
La-14	SAMN02603216	1.99158	34.7	1781	Human
LA-G80-111	SAMN15165794	1.99198	34.7	1788	Unknown
LMG P-21904	SAMN07187785	1.96566	34.6	1780	Commercial Probiotic Products
NCFM	SAMN02603047	1.99356	34.7	1760	Human
P2	SAMN07665576	2.04684	35.7	1904	Commercial Probiotic Products
s-13	SAMN15579847	1.96575	34.6	1778	Unknown
s-4	SAMN15579838	1.95327	34.6	1724	Unknown
UBLA-34	SAMN10136005	1.95104	34.6	1762	Fermented foods
WG-LB-IV	SAMN04628015	1.95169	34.6	1780	Fermented dairy product
YT1	SAMN08142761	2.09254	34.7	1878	Unknown

The underline represented that draft genomes of these strains were sequenced in this study.

**Table 2:** Host Information of 11 New Isolated *L. acidophilus*.

Strain	Age	Gender	Modernization
FAHWH11L56	3	Female	City
FCQHC4LH1	20	Male	Rural
FFJND6L5	24	Male	City
FFJND7L5	24	Female	Rural
FGSYC48L79	NA	NA	Rural
FHNXY41L162	80	Female	Rural
FNMGHHHT12L40	23	Female	City
FSHXBX32L130	59	Female	Rural
FXJSW24L139	2.3	Female	Rural
FXJSW48L59	12	Female	Rural
FZJTZ18L25	79	Male	Rural

**Table 3:** CRISPR-Cas systems and prophages in *L. acidophilus*.

Strain	CRISPR-Cas	Prophage
ATCC 4356	incomplete	incomplete
ATCC 4796	incomplete	incomplete
ATCC 53544	incomplete	incomplete
BIO6307	incomplete	incomplete
CCFM137	incomplete	incomplete
CIP 76.13	incomplete	incomplete
CIRM-BIA 442	incomplete	incomplete
CIRM-BIA 445	incomplete	incomplete
DS10_1A	incomplete	incomplete
DS11_1A	incomplete	incomplete
DS13_1A	incomplete	incomplete
DS13_1B	incomplete	incomplete
DS2_1A	incomplete	incomplete
DS20_1	incomplete	incomplete
DS24_1	incomplete	incomplete
DS5_1A	incomplete	incomplete
DS8_1A	incomplete	incomplete
DS9_1A	incomplete	incomplete
DSM 20079	incomplete	incomplete
DSM 20242	incomplete	incomplete
DSM 9126	incomplete	incomplete
FAHWH11L56	incomplete	incomplete
FCQHC4LH1	incomplete	incomplete
FFJND6L5	incomplete	incomplete
FFJND7L5	incomplete	incomplete
FGSYC48L79	incomplete	intact
FHNXY41L162	complete	intact
FNMGHHHT12L40	incomplete	incomplete
FSHXBX42L130	incomplete	intact
FSI4	incomplete	incomplete
FXJSW24L139	incomplete	incomplete
FXJSW48L59	complete	intact
FZJTZ18L25	incomplete	intact
LA_AVK1	incomplete	incomplete
LA_AVK2	incomplete	incomplete
LA1	incomplete	incomplete
La-14	incomplete	incomplete
LA-G80-111	incomplete	incomplete
LMG P-21904	incomplete	incomplete
NCFM	incomplete	incomplete
P2	incomplete	incomplete
s-13	incomplete	incomplete
s-4	incomplete	incomplete
UBLA-34	incomplete	incomplete
WG-LB-IV	incomplete	incomplete
YT1	complete	intact

## Data Availability

All raw sequencing data analysed in this study have been submitted to the NCBI Sequence Read Archive (https://www.ncbi.nlm.nih.gov/sra/) (accessed on 28 June 2021) under the BioProject PRJNA736624.
